# *Vibiro vulnificus* hemolysin associates with gangliosides

**DOI:** 10.1186/s12866-020-01755-1

**Published:** 2020-03-30

**Authors:** Takashige Kashimoto, Hiroyuki Sugiyama, Keigo Kawamidori, Kohei Yamazaki, Takehiro Kado, Kaho Matsuda, Toshio Kodama, Takao Mukai, Shunji Ueno

**Affiliations:** 1 Laboratory of Veterinary Public Health, School of Veterinary Medicine and Animal Sciences, Kitasato University, Higashi 23-35-1, Towada, Aomori, 034-8628 Japan; 2 Department of Bacterial Infections, International Research Center for Infectious Diseases, Research Institute for Microbial Diseases, Osaka University, Suita, Osaka, Japan; 3 Laboratory of Biomolecular Science, School of Veterinary Medicine and Animal Sciences, Kitasato University, Higashi 23-35-1, Towada, Aomori, Japan

**Keywords:** Gangliosides, Hemolysin, Receptor, *Vibrio vulnificus*

## Abstract

**Background:**

*Vibrio vulnificus* hemolysin (VVH) is a pore-forming toxin secreted by *Vibrio vulnificus*. Cellular cholesterol was believed to be the receptor for VVH, because cholesterol could bind to VVH and preincubation with cholesterol inhibited cytotoxicity. It has been reported that specific glycans such as N-acetyl-D-galactosamine and N-acetyl-D-lactosamine bind to VVH, however, it has not been known whether these glycans could inhibit the cytotoxicity of VVH without oligomer formation. Thus, to date, binding mechanisms of VVH to cellular membrane, including specific receptors have not been elucidated.

**Results:**

We show here that VVH associates with ganglioside GM1a, Fucosyl-GM1, GD1a, GT1c, and GD1b by glycan array. Among them, GM1a could pulldown VVH. Moreover, the GD1a inhibited the cytotoxicity of VVH without the formation of oligomers.

**Conclusion:**

This is the first report of a molecule able to inhibit the binding of VVH to target cells without oligomerization of VVH.

## Background

A wide variety of pathogenic bacteria, both Gram-positive and Gram-negative, produce pore-forming toxins (PFTs) [1–3]. VVH is a PFT secreted by *V. vulnificus* that induces cytotoxicity against variety of cells and cell types by forming small pores on target cell membrane via oligomerization of toxin-monomer [4, 5]. Cholesterol exists in every type of cell ubiquitously and pre-incubation with cholesterol inhibited the cytotoxicity of VVH [6, 7]. For these reasons, cellular cholesterol was believed to be a good candidate cellular receptor for VVH. On the other hand, it is also known that cholesterol induces oligomerization of VVH, and VVH oligomer loses its ability to bind to the target cells [8]. To date, no molecule has been shown to have the ability to inhibit cellular binding of VVH without forming oligomers. VVH is composed of two domains, a β-trefoil lectin domain and a pore-forming domain [5, 9]. Although the β-trefoil lectin domain has carbohydrate binding motif QxW and recognizes N-acetyl-D-galactosamine, (GalNAc) and N-acetyl-D-lactosamine (LacNAc) directly [9], the first accessible domain of VVH to target cell membrane would be the pore-forming domain, according to an analysis of the three-dimensional structure of this toxin [5]. Thus, the function of carbohydrates and cellular cholesterol in the binding mechanism of VVH to cellular membrane has remained controversial. In this study, we found that cellular cholesterol is not necessary for the binding of VVH to target cells. Gangliosides associates with the VVH directly and inhibit the cytotoxicity of VVH without oligomerization. This is the first report of a molecule that can inhibit the binding of VVH to the cellular membrane without oligomer formation.

## Result

### Cellular cholesterol is not a receptor for VVH

**Fig. 1:**
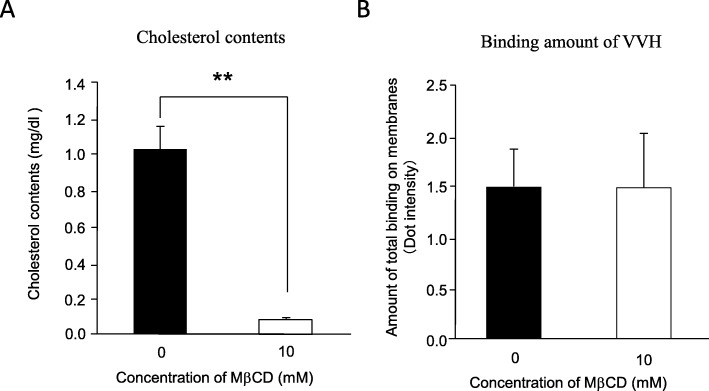
Cholesterol depletion has no effect on binding of VVH (**a**) Measurement of cholesterol contents both in cholesterol depleted and non-depleted ghosts. **b** Binding amount of VVH both on cholesterol depleted and non-depleted ghosts. Data are presented as means ± S.D. and represent three independent experiments, each in triplicate samples. **; Significant decrease compared with the cholesterol content in MβCD-untreated ghosts (ANOVA and Tukey’s test, *P* < 0.01)

VVH targets and lyses a wide variety of cells such as epithelial cells, fibroblasts, endothelial cells, and erythrocytes [11–14]. Cellular cholesterol is thought to be a good candidate receptor of VVH because its components are ubiquitously expressed on cellular membranes in mammalian cells. In our previous study, although the percentage of cellular cholesterol was decreased to 36.3 ± 4.3% of the control in 8 mM Methyl-β-cyclodextrin (MβCD)-treated HeLa cells, the amount of VVH binding in 8 mM MβCD-treated HeLa cells only decreased to approximately 60% [7]. To demonstrate the involvement of cellular cholesterol in the binding of VVH to cellular membrane more clearly, cellular cholesterol was depleted in various types of cells using higher concentrations of MβCD. However, such higher concentrations of MβCD treatment itself induced cell death since cellular cholesterol was essential to maintain membrane stability (data not shown). In this study, we finally succeeded in achieving advanced depletion of cellular cholesterol in a ghost membrane that was prepared from bovine erythrocytes. Cholesterol contents of erythrocyte ghosts was decreased from 1.03 ± 0.1 mg/dl to 0.1 ± 0.0 mg/dl by treatment with 10 mM MβCD, whereas the VVH binding on 10 mM MβCD-treated erythrocyte ghosts was not decreased compared with that of MβCD non-treated ghost membrane (Fig. 1). These data clearly indicated that cellular cholesterol is not a receptor for VVH on target cells.

### Gangliosides inhibit cytotoxicity by preventing the binding of VVH to the cells

**Fig. 2:**
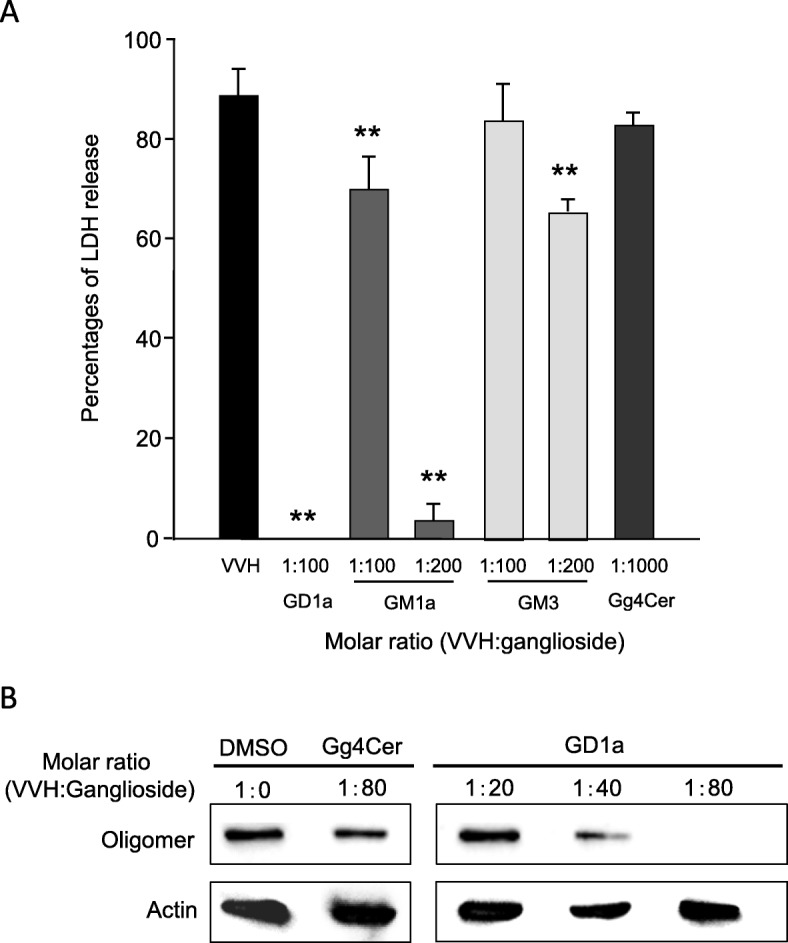
Gangliosides inhibit cytotoxicity by preventing VVH binding. **a** Inhibition of VVH-induced cytotoxicity by various gangliosides. VVH were preincubated with various gangliosides at the indicated molar ratio (VVH:ganglioside). The percentages of LDH release were calculated as described in the Materials and Methods. **; Significant decrease compared with the LDH release of VVH without ganglioside (ANOVA and Tukey’s test, P < 0.01). **b** Prevention assay for binding of VVH on CHO cells by ganglioside. VVH was preincubated with Gg4Cer or GD1a as the indicated molar ratio. Binding of VVH and cellular actin were detected by using appropriate antibodies as described in Materials and Methods

It was reported that VVH binds to GalNAc and LacNAc by β-trefoil domain [9]. Therefore, first we analyzed the inhibition effect of simple sugars and glycans on VVH cytotoxicity. Glucose, Galactose, GalNAc, Lactose and Dextrose were tested. Only GalNAc could inhibit the cytotoxicity of VVH, however, about 1,000,000-fold of GalNAc in molar ratio against VVH was needed to inhibit the cytotoxicity of VVH by 95% (data not shown). We considered that an additional component would be needed for more effective inhibition of VVH cytotoxicity. Next, we tried to inhibit the VVH cytotoxicity by pre-incubation with various gangliosides (glycolipid). As shown in Fig. 2a, the VVH was highly cytotoxic (88.7 ± 5.2%) against Chinese hamster ovary (CHO) cells, but this cytotoxicity was completely inhibited by pre-incubation with ganglioside GD1a (100-fold molar ratio against VVH, 0%). Also, GM1a (200-fold, 4.0 ± 3.2%), and GM3 (200-fold, 65.3 ± 2.3%) could inhibit the cytotoxicity of VVH, but Gg4Cer could not, even after pre-incubation with 1000-fold of VVH in molar ratio (Fig. 2a). Gg3Cer, some globosides, fetuin (sialylated N-linked and O-linked glycoprotein) and transferrin (N-linked glycoprotein) were also examined but could not inhibit the VVH cytotoxicity (data not shown). All the gangliosides, which could inhibit the cytotoxicity of VVH, have neuraminic acid in their structure, but not Gg4Cer, a ganglioside that could not inhibit the cytotoxicity of VVH. Therefore, we tried to inhibit the cytotoxicity of VVH by pre-treatment of neuraminidase to CHO cells. However, the pre-treatment by 100 mU of neuraminidase on CHO cells could not inhibit the cytotoxicity of VVH (data not shown). VVH probably recognize more complex structure of glycan.

It has been reported that VVH binds to cellular membranes as a monomer, and then forms an oligomer. To determine whether the ganglioside GD1a could inhibit the binding of VVH to CHO cells or not, CHO cells were exposed to mixtures containing VVH and various molar ratios of GD1a or Gg4Cer for 1 h at 37 °C. All the bound VVH could oligomerize under these conditions. A shown in Fig. 2b detection of VVH oligomer decreased in a concentration dependent manner after treatment with GD1a, but not with Gg4Cer. VVH monomer could not be detected even when the oligomer formation was inhibited by GD1a. These data indicated that GD1a effectively inhibits the cytotoxicity of VVH by preventing the binding of VVH to the CHO cells.

### GD1a did not induce oligomer formation of VVH

**Fig. 3:**
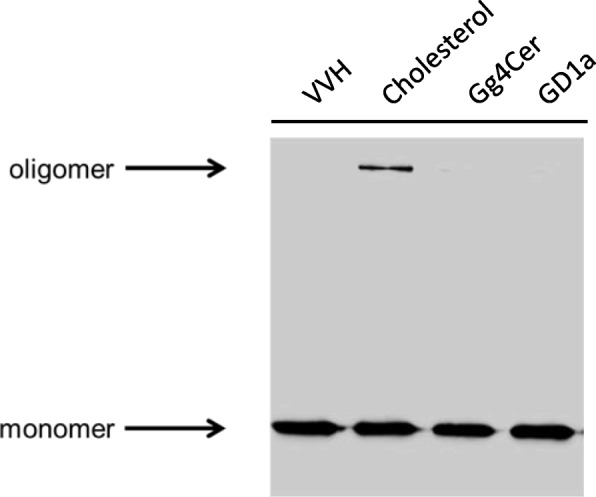
Oligomerization assay. VVH were incubated with cholesterol, Gg4Cer or GD1a at a molar ratio of 1:100 for 30 min at 37 °C individually, and were subjected to SDS-PAGE followed by Western blotting with anti-VVH polyclonal antibody

GD1a inhibited the binding of VVH to target cells. Although it is well known that cholesterol also could inhibit the cytotoxicity of VVH, it induces the conversion of monomer to oligomer in VVH [7, 8]. We investigated whether GD1a induces oligomer formation of VVH or not. VVH were pre-incubated with cholesterol, Gg4Cer, or GD1a for 1 h at 37 °C, and both the monomer and the oligomer of VVH were examined in these mixtures by western blotting using anti-VVH antibody. As shown in Fig. 3, cholesterol induced oligomer formation in VVH, whereas GD1a did not. Thus, GD1a is the first discovered molecule which can inhibit the binding of VVH to target cells without oligomer formation in VVH. Cellular cholesterol might be a trigger factor for conversion from monomer to oligomer after binding of this toxin to the membrane of target cells.

### Certain gangliosides directly bind to VVH

**Fig. 4:**
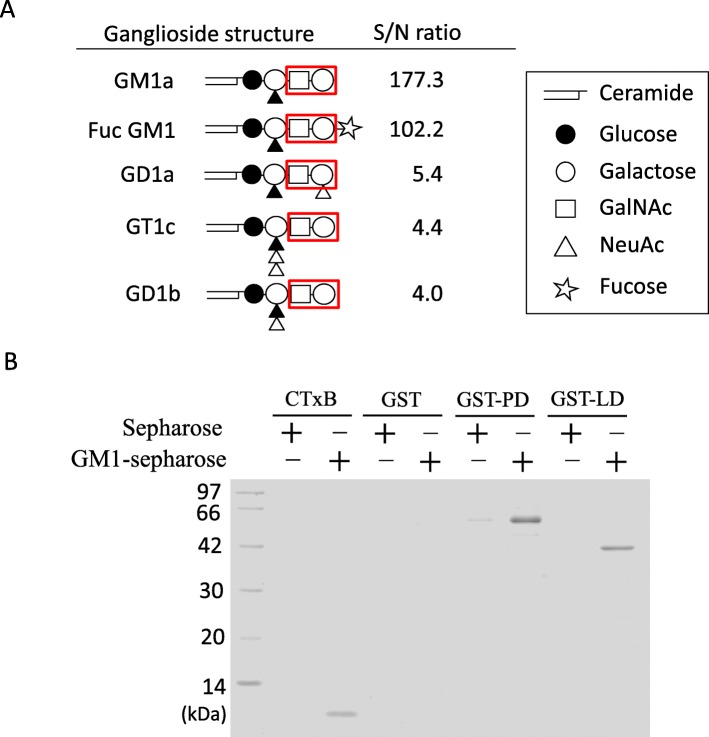
VVH bind to gangliosides. **a** The structures of gangliosides that associate with VVH by glycolipid array. The red polygons indicate minimum structure for binding to VVH. **b** Pull-down assay by Lyso-GM1 sepharose. The pore-forming domain (PD) and lectin domain (LD) of VVH were expressed as the GST-fusion protein. Both domains of VVH, GST alone and the CTx-B were tried to pulled down with GM1 coupling sepharose. The GST-PD protein (21–300 a.a. of VVH without 20 a.a. of signal sequence) and GST-LD protein (301–451 a.a. of VVH) were expressed as the 59 kDa and the 43 kDa GST-fusion protein respectively

To gain more insight into the binding mechanism of VVH to gangliosides, we examined which gangliosides associate with VVH. VVH was applied onto a glycolipid array with various gangliosides on a glass chip, and VVH binding was detected by streptoavidin-Cy5. Positive binding was determined according to the manufacturer’s instructions (S/N ratio more than 3.0). This assay showed that VVH bound directly to GM1a, FucosylGM1, GD1a, GT1c, and GD1b (Fig. 4a). The strength of the binding of these gangliosides to VVH was in the order GM1a > FucGM1 > > GD1a > GT1c > GD1b (Fig. 4a). On the other hand, VVH did not associate with GT1a, GM2, GM3, GT3, GD2, GT2, GM1b, Gg4Cer, and Gg3Cer specifically (S/N less than 3.0). All the gangliosides that associate with VVH possessed Galβ1-3GalNAc as a minimum common structure (Fig. 4a). It was reported that VVH was composed of two domains, pore-forming domain (PD) and lectin domain (LD) [5, 9]. We expressed the full length of VVH and the both PD and LD of VVH by using *Eschelichia coli* protein expression system as the glutathione S-transferase (GST) fusion protein. The enough proteins of GST-PD and the GST-LD could be expressed and purified, but the full length of VVH (GST-VVH) was not, due to the formation of inclusion body unfortunately. Cholera Toxin B subunit (CTx-B) is known to bind many glycans and glycoconjugates including GM1, GM2, GD1a, GM3, Toll-like receptor 4 Fc, Triggering receptor expressed on myeloid cells 2, Leukocyte mono-immunoglobulin–like receptor 5, and so on [15]. To confirm the direct binding of VVH to the gangliosides, we performed the pull-down assay of VVH using lyso-GM1 sepharose. The purified GST-PD, the purified GST-LD, the CTx-B, and the purified GST was mixed with lyso-GM1 sepharose, and tried to pull-down by using lyso-GM1 sepharose. The GST-PD, GST-LD, and the CTx-B pulled down with GM1 coupling sepharose, but not GST (Fig. 4b). These data obviously showed that VVH directly binds to GM1. It will be necessary to further analyze, whether the binding of both domains against ganglioside will be needed for the cytotoxicity of VVH.

## Discussion

Pore forming toxins make small ion permeable pores on the target cellular membrane by drastically changing their structure after binding to the target cellular membrane [16–18]. However, to date, the detailed mode of action of VVH has not been elucidated. In this study, we proposed that certain gangliosides associate with VVH directly, and that cellular cholesterol might be a converting factor from monomer to oligomer. The amount of VVH binding to the ghost membrane was not affected by cholesterol depletion (Fig. 1). This result demonstrated that the cellular cholesterol is not the cellular receptor for VVH on target cells suggested that other molecules must be involved. Although, it was reported that the treatment of cells with MβCD also removes other molecules except for cholesterol including gangliosides and leading to a dynamic remodeling of membrane complex lipids [19], the binding amount of VVH on the ghost membrane was not affected (Fig. 1). There are the possibilities that enough number of molecules with Galβ1-3GalNAc left on the membrane or cellular receptor might be different depend on a cell type. In a previous study, the glycan specificity of binding for the recombinant β-trefoil lectin domain of VVH was analyzed using glycan array [9]. The β-trefoil lectin domain mostly recognized the Galβ1–4GlcNAc and Galβ1-3GalNAc [9]. Our study found that all the gangliosides that associate with VVH harbor the Galβ1-3GalNAc as the common structure (Fig. 4a). Thus, the Galβ1-3GalNAc was thought to be the minimum structure from both this and previous studies [9]. Moreover, the Galβ1-3GalNAc-harboring gangliosides including GD1a, GM1a and GM3 could inhibit the cytotoxicity of VVH (Fig. 2a and b). Among them, the GD1a inhibited the binding of VVH to the target cells without oligomer formation (Fig. 2b and 3), and the GM1 could pulldown the VVH in our study (Fig. 4b) These results suggested that the Galβ1-3GalNAc-harboring gangliosides and other molecules with Galβ1-3GalNAc might be one of a cellular receptor for VVH. This is the first report of a molecule that can inhibit the binding of VVH without oligomer formation.

**Fig. 5:**
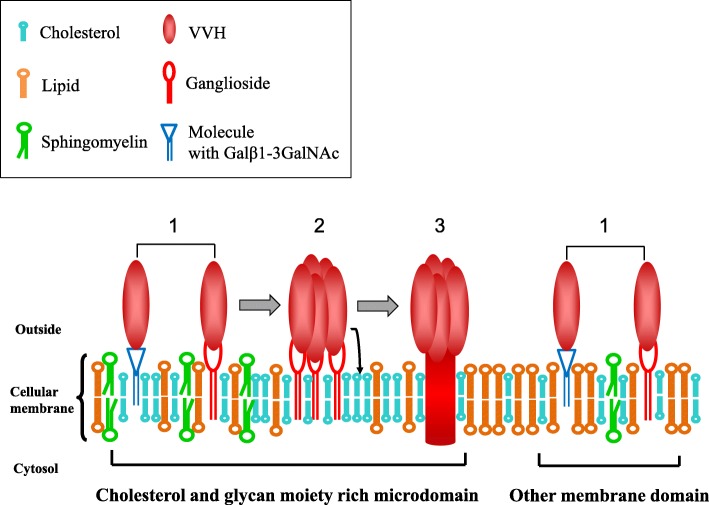
Speculative cartoon representation of VVH toxic steps on cellular membrane. Step 1; Binding to ganglioside (Galβ1-3GalNAc) and to unknown molecule with Galβ1-3GalNAc at both cholesterol and glycan rich micro domain, and other membrane domain. Step 2; Interaction with cholesterol for oligomerization. Step 3; Conformational change to pore-form. Step 2 and 3 occurs at only cholesterol and glycan rich micro domain

It is well known that cholesterol could inhibit the binding of VVH to target cells, but it induces oligomer formation of VVH. These facts suggested that cholesterol may be a trigger factor for conformational change of VVH from membrane binding form to pore-form on the target cellular membrane. In fact, VVH localized at membrane regions which are relatively abundant in cholesterol in our previous report (Fig. 5) [7]. In addition, *Vibrio cholerae* hemolysin/cytolysin (HlyA/VCC), which has a similar structure to VVH, binds to complex N-glycan [20], and it was reported that oligomer formation of HlyA/VCC was drastically accelerated by cholesterol in a lipid bilayer [21, 22]. These toxins might have a similar mode of action with VVH in cellular intoxication.

In this study, we showed that VVH associates with the gangliosides, which are harbouring the Galβ1-3GalNAc as a minimum structure (Fig. 4b). On the other hand, N-linked and O-linked glycoproteins such as fetuin and transferrin, and some glycolipids (Gg2Cer, and some globosides) could not inhibit the VVH cytotoxicity (data not shown). From these data and our previous report [7] suggested that VVH localized on both cholesterol and glycan moieties rich membrane domain and other membrane domain (Fig. 5). VVH firstly accesses both membrane domains by association with the specific glycans with Galβ1-3GalNAc, and then may convert from monomer to oligomer to form an ion permeable pore by recognizing the cellular cholesterol only at cholesterol and glycan moieties rich micro domain (Fig. 5). Unfortunately, we could not indicate the importance of gangliosides in cellular level in this study. Further studies are needed whether the certain gangliosides are the receptor of VVH or not.

## Conclusions

In conclusion, we found in this study that VVH might recognize the certain molecules, which have Galβ1-3GalNAc in their component at both cholesterol and glycan rich microdomain, and other membrane domain, then triggering the oligomerization by interaction with cholesterol only on cholesterol and glycan rich microdomain for pore-formation.

## Methods

### Cell culture

Chinese hamster ovary (CHO) cells were grown in Dulbecco’s modified Eagle’s minimum essential medium (DMEM; Gibco BRL Life Technologies, Rockville, MD) supplemented with 2 mM glutamine, 2 mM sodium pyruvate, and 10% heat-treated fetal calf serum. Cells were incubated at 37 °C under 5% CO2 in air in a humidified atmosphere.

### Reagents, Gangliosides and antibodies

Gangliosides, Methyl-β-cyclodextrin (MβCD) and Cholesterol were purchased from Sigma (St. Louis, MO). Cellular actin was analyzed by western blotting with anti-actin monoclonal antibody, clone C4 (Chemicon International Inc., Temecula, CA). VVH was detected by using anti-VVH polyclonal antibody, which was produced as described previously (5).

### Preparation of VVH

VVH was purified from the culture supernatant of the *V. vulnificus* K1 strain as described previously [10]. The VVH was purified with HiLoad 16/10 Phenyl-sepharose (GE healthcare., Boston MA). The purity of the VVH was confirmed by sodium dodecyl sulfate polyacrylamide gel electrophoresis (SDS-PAGE) with staining solution containing 0.5% Coomassie brilliant blue R-250. The highly purified VVH containing fractions were dialyzed in 10 mM glycine buffer (pH 9.8)–150 mM NaCl at 4 °C for 16 h. These fractions were pooled and used as the purified VVH for this study. The specific activity of purified VVH was confirmed by examining the hemolytic activity against mouse erythrocytes (> 70,000 hemolytic units/mg of protein).

### Preparation of ghost membrane

Bovine defibrinated blood was suspended in hemolysis buffer (5 mM Na_2_HPO_4_, pH 8.0) for 10 min, on ice. Cells were then centrifuged at 12,000×g for 10 min. The pellet was washed several times until the color changed to white in hemolysis buffer. After hemolysis, the ghosts were kept in storage buffer (140 mM NaCl, 20 mM Tris-HCl pH 7.5), and were used for toxin binding assay and measurement of cholesterol contents.

### Measurement of cholesterol contents

Cholesterol contents of both 10 mM MβCD treated- and untreated ghost membranes were assayed by a Cholesterol E-Test Wako (Wako, Osaka, Japan). Briefly, ghost membranes were treated with 10 mM MβCD, and then washed twice with 1 ml of cold PBS. After washing with cold PBS, the ghost membranes were lysed with lysis buffer. Six hundred fifty microliters of the lysate was mixed with 100 μl of the cholesterol assay kit buffer. This mixture was further mixed with 750 μl of concentration enzyme mix solution, then incubated for 5 min at 37 °C prior to measuring absorbance at 600 nm. The cholesterol contents were calculated as follows: (measured fluorescence of sample /fluorescence of standard cholesterol) × 200. The percentage of remaining cholesterol after pretreatment with MβCD was determined as follows: (measured fluorescence of treated cells obtained from a standard curve/total fluorescence of untreated cells) × 100.

### Measurement of binding amount of VVH

The ghost membranes were treated by 10 mM of MβCD for 30 min at 37 °C, and then washed twice with 1 ml of cold HBSS. After washing, the ghost membranes were incubated with 5 μg/ml of VVH for 30 min at 37 °C. The ghost membranes were centrifuged at 8000×g, and washed twice with storage buffer. After washing, the cells were lysed by lysis buffer (24.7 mM Tris pH 8.3, 192 mM glycine, 20% v/v methanol). The bound VVH and cellular actin were detected by dot blotting using antibodies against anti-VVH and anti-actin. The dot intensities of these proteins were measured using NIH Image J software. Amount of bound VVH was calculated by dividing the dot intensity of VVH by that of actin.

### Cytotoxicity assays

Cytotoxic activity was measured by using a Lactate dehydrogenase (LDH) release as the previously described (5). Briefly, cells were seeded in 24-well tissue-culture plates at 1 × 10^5^ cells/well and incubated for 24 h. The cells were washed with HBSS, and then replaced with pre-warmed DMEM. The VVH and various gangliosides were pre-incubated at indicated molar ratio for 30 min at 37 °C. The mixture was inoculated into the wells and incubated for 2 h at 37 °C, then aliquots of medium samples (sample LDH) were assayed for LDH activity. Cells treated with VVH vehicle only (control LDH) were used to calculate background LDH activity, and cells lysed with 0.5% TritonX-100 were used to represent total LDH activity. The percentage LDH release was calculated as (sample LDH – control LDH) / (total LDH – control LDH) × 100.

### Prevention assay for binding of VVH on CHO cells

CHO cells were seeded in 6-well tissue-culture plates at 5 × 10^5^ cells/well. After 48 h, the cells were washed twice with HBSS, and then replaced with DMEM. The mixture was pre-incubated with the VVH and GD1a or Gg4Cer at the indicated molar ratio, inoculated into the wells, and then incubated for 1 h at 37 °C. During this incubation time, all the VVH that bound to CHO cells were oligomerized. After washing three times with HBSS, the cells were extracted with lysis buffer supplemented with 1% Triton X-100 and a protease inhibitor mixture. Bound VVH and cellular actin were detected by western blotting using antibodies against anti-VVH and anti-actin..

### Oligomerization assay

VVH were incubated with cholesterol, Gg4Cer or GD1a for 30 min at 37 °C. The mixture of VVH and ganglioside was subjected to SDS-PAGE followed by western blotting using anti-VVH polyclonal antibody.

### Glycolipid array

The glycolipid array assay was performed using a glycolipid array plate (Sumitomo Bakelite, Tokyo, Japan). VVH was adjusted to 100 μg/mL in a reaction buffer comprising 50 mm Tris/HCl, pH 7.5, 100 mm NaCl, 1 mm CaCl_2_, 1 mm MgCl_2_, 1 mm MnCl_2_ and 0.05% Tween-20. The glycolipid array plates were incubated with VVH for 2 h at room temperature. After washing sequentially with a washing buffer (50 mm Tris/HCl, pH 7.4, 100 mm NaCl) and water, the plates were incubated with biotin conjugated anti-VVH polyclonal IgG, and subsequently probed with streptoavidin-Cy5 (Jackson Immunoresearch). The fluorescent signal was measured using a ScanArray Express Version4.0 (Perkin-Elmer, Waltham, MA, USA). The binding signal is measured by Cy5 fluorescence, and the data is expressed as signal / noise (S / N) values. The S / N values ​​are calculated by dividing the fluorescence intensity of each spot by the background intensity three times and are expressed as the average intensity of those measurements. S/N values > 3 were considered to indicate significant binding of the VVH to glycolipids.

### Construction, expression, and purification of GST-fusion protein

The *V. vulnificus* genome DNA was purified by Qiagen Genomic-tip (Qiagen, Hilden, Germany) as recommended by the manufacturer. VVH encoding gene, *vvhA* was amplified with signal sequence by PCR with the primers vvhA5’ (5′-GTGGGATCCATGAAAAAAATGACTCTGTTTACC-3′;the underline indicates an *Bam*HI site) and the vvhA3’(5′-GTGGCATGCCTAGAGTTTGACTTGTTGTAATGT − 3′; the underline indicates an *Sph*I site), from *V. vulnificus* genome as the template. The amplified DNA was ligated to pGEM-T vector (Promega, Madison, WI) and the sequence was confirmed by DNA sequencing. GST-PD and GST-LD were amplified by using the following primer pairs from pGEMT *vvhA* as the template respectively. GST-PD FW; 5′-GGATCCGTGAAACAACGTATTCGCATCGAC-3′ (the underline indicates an *Bam*HI site), GST-PD Rev.; 5′-CTCGAGCTAGAGTTTGACTTGTTGTAATGT-3′ (the underline indicates an *Xho*I site), and GST-LD Fw; 5′-GGATCCCAAGAATATGTGCCGATTGTTGAG-3′ (the underline indicates an *Bam*HI site), GST-LD Rev.; 5′-CTCGAGCTAGGTACTGCTGGTTGACGAGCC-3′ (the underline indicates an *Xho*I site). The amplified each DNA was ligated to pGEM-T vector and the sequence was confirmed by DNA sequencing, and then ligated to pGEX4T3 (GE Healthcare Life Sciences, Chicago, IL) *Bam*HI-*Xho*I site. Each plasmid was transformed to *Escherichia coli* DH5α. The bacteria were cultivated in Luria-Bertani (LB) broth containing 100 μg of Ampicilin/ml until OD _600_ 0.5 at 37 °C and then induced to produce the GST-fusion protein by adding 0.1 mM isopropyl-β-D-thiogalactopyranoside (IPTG) at 20 °C for 16 h. After induction of the protein, the bacteria were suspended with the binding buffer (1 mM EDTA, 50 mM NaCl, 50 mM Tris-HCl pH 8.0). The bacterial suspension was sonicated using a Vibra Ultrasonic (model VCX-500, Sonics and Materials Inc., USA) and centrifuged at 21,000×g at 4 °C for 20 min. The supernatant was used for purification of GST-fusion proteins (GST-PD and GST-LD). The GST-fusion protein was purified with Glutathione Sepharose 4B Resin according to the manufacturer’s instructions (GE Healthcare Life Sciences, Chicago, IL). After purification, each GST-fusion protein was dialyzed with phosphate buffered saline (PBS). The GST-PD protein (21–300 a.a. of VVH without 20 a.a. of signal sequence) and GST-LD protein (301–451 a.a. of VVH) were expressed and confirmed as the ca. Fifty nine kDa and the ca. Forty three kDa GST-fusion protein respectively by SDS-PAGE.

### Pull-down assay by GM1-Sepharose

Pull-down assay by using GM1-Sepharose for the toxins were performed as previously described [23]. Briefly, lyso-GM1 was coupled using NHS-activated Sepharose 4 Fast Flow (GE Healthcare, England) in 0.2 M NaHCO_3_ and 0.5 M NaCl (pH 8.3) at room temperature for 4 h with rotation. After the coupling reaction, non-reacted groups on the Sepharose were blocked by 0.5 M ethanolamine in the coupling solution. Lyso-GM1 Sepharose was then washed with 0.1 M Tris–HCl, 0.1 M acetate and 0.5 M NaCl and resuspended with PBS in a 1:1 (volume/volume) ratio. GST-LD, GST-PD, GST, or CTxB was incubated with lyso-GM1 or lyso-GM1 non-coupling Sepharose (control Sepharose) in PBS for 2 h at 4 °C with rotation. After incubation, Sepharose was sedimented by centrifugation at 12,000×*g*. The supernatant was discarded, and the remaining Sepharose was washed twice with PBS. The bound proteins were then solubilized with sample buffer (62.5 mM Tris–HCl, 2% SDS, 10% glycerol, 0.001% bromophenol blue and 100 mM dithiothreitol) and boiled for 5 min. The sample was analyzed by SDS-PAGE and visualized by 0.5% *Coomassie Brilliant Blue R-250*.

## Data Availability

The datasets used and/or analysed during the current study available from the corresponding author on reasonable request.

## Abbreviations

CHO: Chinese hamster ovary
CTx-B: Cholera Toxin B subunit
FucGM1: Fucα1-2Galβ1-3GalNAcβ1–4(Neu5Acα2–3) Galβ1–4Glc-Cer
GalNAc: N-acetyl-D-galactosamine
Gg3Cer: βDGalpNAc(1–4)βDGalp(1–4)βDGlcp(1–1)Cer
Gg4Cer: βDGalp(1–3)βDGalpNAc(1–4)βDGalp(1–4)βDGlcp(1–1)Cer
GD1a: Neu5Acα2–3Galβ1-3GalNAcβ1–4(Neu5Acα2–3)Galβ1–4Glc-Cer
GD1b: Galβ1-3GalNAcβ1–4(Neu5Acα2-8Neu5Acα2–3)Galβ1–4Glc-Cer
GD2: GalNAcβ1–4(Neu5Acα2-8Neu5Acα2–3)Galβ1–4Glc-Cer
GM1a: Galβ1-3GalNAcβ1–4(Neu5Acα2–3)Galβ1–4Glc-Cer
GM1b: Neu5Acα2–3Galβ1-3GalNAcβ1–4Galβ1–4Glc-Cer
GM2: GalNAcβ1–4(Neu5Acα2–3)Galβ1–4Glc-Cer
GM3: Neu5Acα2–3Galβ1–4Glc-Cer
GST: Glutathione S-transferase
GT1a: Neu5Acα2-8Neu5Acα2–3Galβ1-3GalNAcβ1–4(Neu5Acα2–3)Galβ1–4Glc-Cer
GT1c: Galβ1-3GalNAcβ1–4(Neu5Aca2-8Neu5Aca2-8Neu5Aca2–3)Galβ1–4Glc-Cer
GT2: GalNAcβ1–4(Neu5Acα2-8Neu5Acα2-8Neu5Acα2–3)Galβ1–4Glc-Cer
GT3: Neu5Acα2-8Neu5Acβ2-8Neu5Acα2–3Galβ1–4Glc-Cer
HlyA/VCC: *Vibrio cholerae* hemolysin/cytolysin
IPTG: Isopropyl-β-D-thiogalactopyranoside
LacNAc: N-acetyl-D-lactosamine
LD: Lectin domain
MβCD: Methyl-β-cyclodextrin
PD: Pore-forming domain
PFTs: Pore-forming toxins
SDS-PAGE: Sodium dodecyl sulfate polyacrylamide gel electrophoresis
S/N: Signal / noise
VVH: *Vibrio vulnificus* hemolysin
